# Grayanotoxin poisoning following fresh rhododendron flower ingestion: a rare case report from rural Nepal

**DOI:** 10.1186/s12245-025-00999-w

**Published:** 2025-09-17

**Authors:** Ashal Timalsina, Arjun Gaire, Roshan Acharya, Anupa Subedi, Rojee Shrestha, Aayusha Suwal

**Affiliations:** Pashupati Chaulagain Memorial Hospital, Dolakha, Nepal

**Keywords:** Grayanotoxin, Rhododendron, Bradycardia, Intoxication

## Abstract

**Background:**

Rhododendron, the national flower of Nepal, is widely used for culinary and traditional medicinal purposes. However, certain species, including *Rhododendron decorum*, contain grayanotoxins known to cause potentially life-threatening toxicity. Toxicity following fresh flower consumption has not been reported.

**Case presentation:**

We report the case of a 58-year-old man who presented with dizziness, headache, nausea and vomiting following the ingestion of four fresh white Rhododendron flowers during a high-altitude visit to Kalinchowk, Nepal. Examination findings revealed hypotension and bradycardia. Supportive treatment with intravenous fluids led to hemodynamic stabilization. On the second day of admission, the patient experienced transient cerebellar symptoms, which resolved without specific intervention. He recovered fully within 48 h.

**Conclusion:**

While the prognosis is generally favorable with supportive care, recognition of characteristic symptoms is essential. This case highlights the risk of Rhododendron toxicity following ingestion of its flowers, even in small amounts. Public education and clinician awareness are crucial to prevent and manage such toxic exposures effectively.

## Background

Poisoning is the process of detrimental effect that occurs following exposure to foreign chemicals substances [1]. Poisoning, either intentional or unintentional, is one of the common presentations in hospital. As of 2021, there is 0.7 deaths per 100k population due to unintentional poisoning worldwide. The number is significantly higher in the context of Nepal, 2.9 deaths per 100k [2]. 

Rhododendron, known as *lali guras* in Nepali, is the national flower of Nepal. About 33 species of Rhododendron have been reported in Nepal. The flowers, along with their beauty are also valued for their culinary and medicinal uses. In Nepal, rhododendron flowers are used to flavor sweet drinks, eaten with salt and chilies, and even added to fish curries in the belief that they help dissolve fish bones. Traditionally, they are also believed to alleviate symptoms of mountain sickness It is also used as traditional medical remedy for cough, joint pain and swelling [3]. 

Rhododendron ingestion is known to cause toxicity. Intoxication has been reported following its ingestion in the form of honey, rarely dried flower and medicinal preparation [4–6]. However, to the best of our knowledge, no cases of grayanotoxin intoxication following the ingestion of fresh rhododendron flowers have been reported in the literature. Here we present a case of rhododendron toxicity in a man following ingestion of fresh flowers.

## Case presentation

A 58-year-old man, a resident of Bhimeshwor (1554 m) visited Kalinchowk (at height of 3842 m). He had consumed four rhododendron flowers. Two hours following ingestion, he developed dizziness, headache, nausea and two episodes of vomiting and presented to emergency department of Dolakha district hospital. He had known comorbidities of hypertension and type 2 diabetes mellitus. He had no history of loss of consciousness, seizures and diarrhea. He had previous visits to similar heights but had never developed such symptoms.

On examination, patient was well oriented to time, place and person with Glasgow Coma Scale (GCS) of 15/15. His blood pressure was 80/50 mmHg, pulse 52 beats/minute (regular), respiratory rate 22/minute and oxygen saturation of 96% in room air. There was no murmur, gallop. Chest auscultation revealed bilateral vesicular breath sounds without any added sounds. The abdomen was soft and non-tender. No neurological abnormality was detected.

**Fig. 1:**
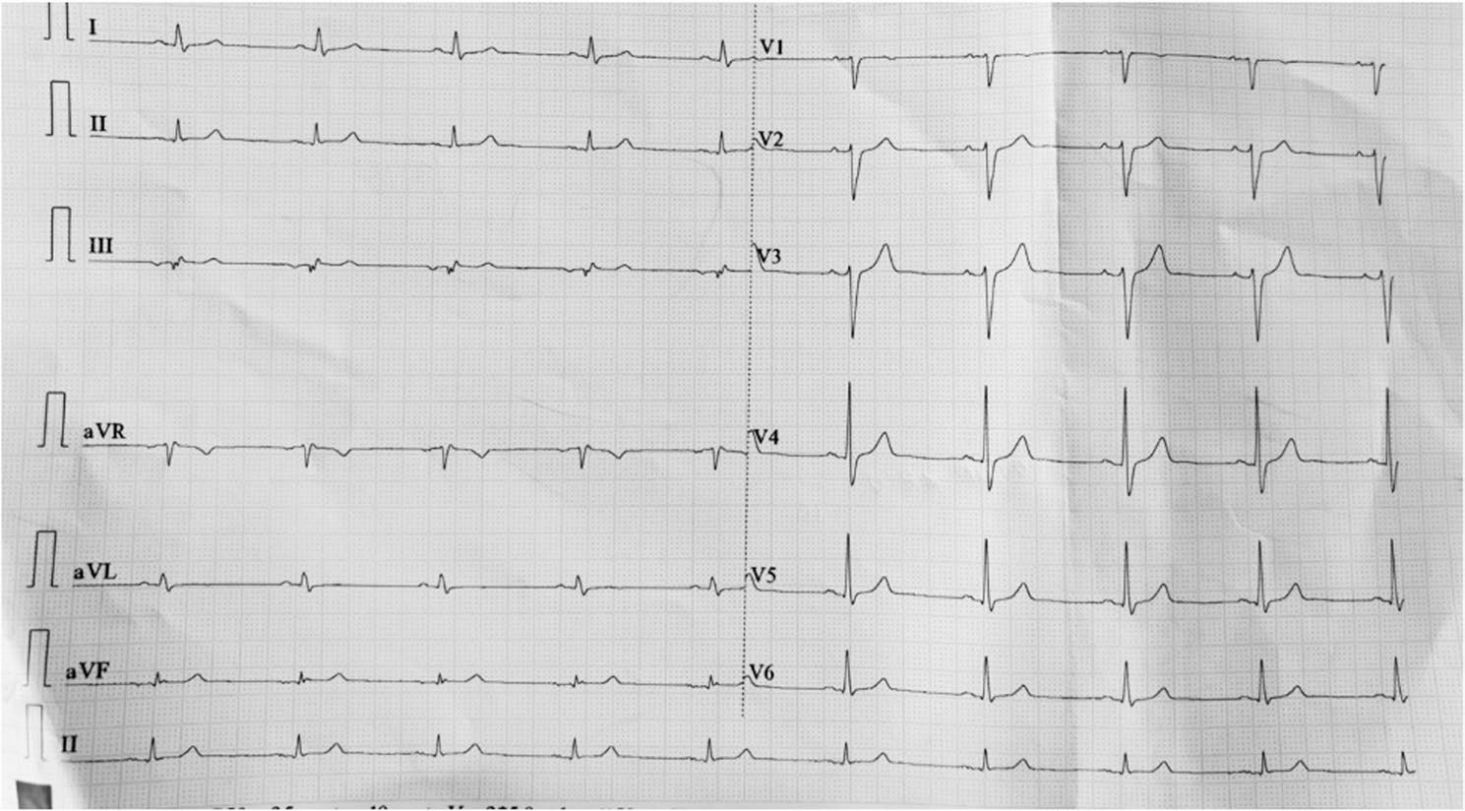
ECG at presentation showed sinus bradycardia with heart rate of 54 bpm, qrs = 100 ms, corrected QT (Rautaharju) = 403 ms

**Fig. 2:**
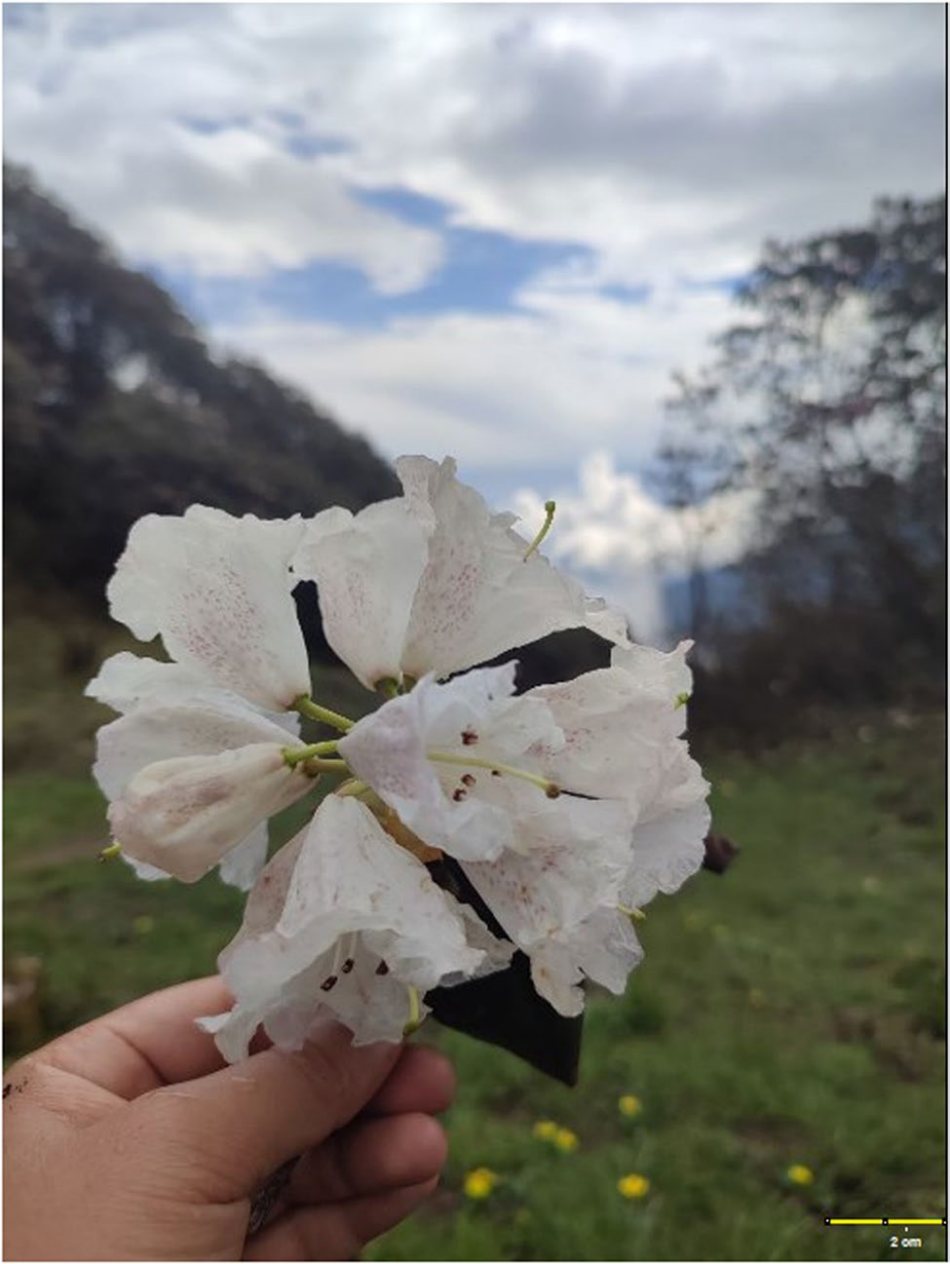
Rhododendron decorum flower

A 12 lead ECG showed sinus bradycardia with heart rate of 58 bpm (Fig. 1: ECG at presentation showed sinus bradycardia with heart rate of 54 bpm, qrs = 100 ms, corrected QT (Rautaharju) = 403 ms). Blood investigations (complete blood counts, renal function tests, liver function tests, random blood glucose) and urine analysis were unremarkable (Table 1: Summary of investigation findings of the patient). His attendant had the pictures of ingested flowers taken during the trip and the flower was identified to be Rhododendron decorum (Fig. 2: Rhododendron decorum flower) [3] that is commonly called “seto guras” in Dolakha district of Nepal.

**Table 1:** Summary of investigation findings of the patient

Test Parameters	Patient’s value	Normal Range
Hemoglobin (gm/dL)	14.5	13.5–17
WBC (/micro liter)	11.82	4–11 * 10^3^
Platelets(/ml)	195	150–450 * 10^3^
Random blood sugar (mg/dl)	96	70–140
Urea/Creatinine (mg/dl)	35/0.8	15–45/0.2–1.3
Na/K (mEq/L)	138/4.7	135–145/3.5–5.5
AST/ALT(IU/L)	32/38	5–40
Bilirubin, Total/Direct (mg/dl)	1.1/0.2	0.1–1.2/<0.3

A provisional diagnosis of rhododendron toxicity was made with acute mountain sickness (AMS), diabetic ketoacidosis (DKA) considered as differentials. Normal lab values ruled out DKA. Presence of bradycardia suggested AMS was less likely.

**Fig. 3:**
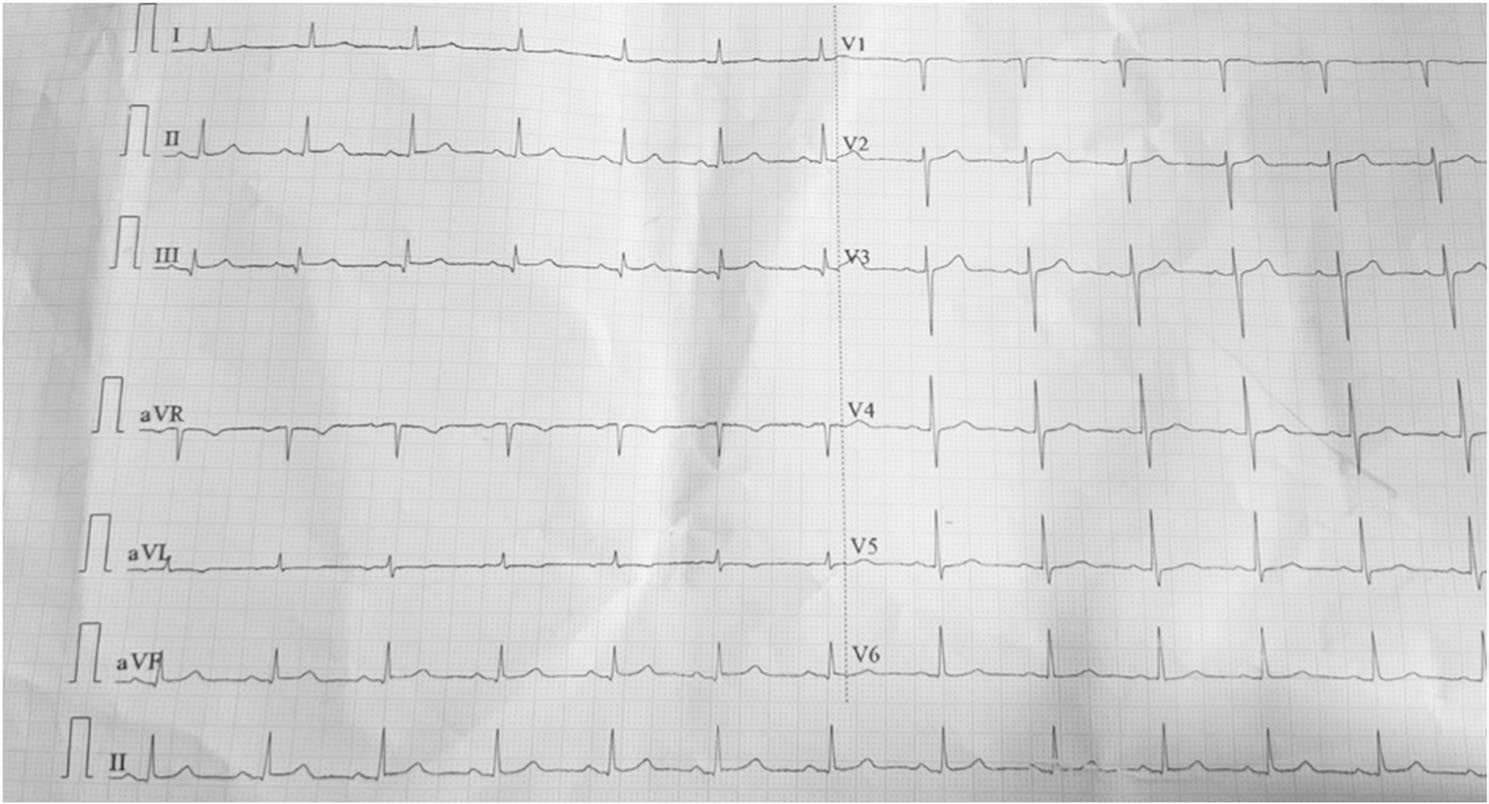
ECG after 48 h of admission showed normal sinus rhythm with heart rate of 78 bpm, qrs = 100 ms, corrected QT (Rautaharju) = 423 ms

Initially, he was resuscitated with 3pints (1500 ml) of normal saline over 30 min. He responded well, blood pressure increased to 100/70mmHg and pulse was 64 bpm. Following stabilization, he was shifted to ward for close monitoring. Maintenance fluid was continued. Antihypertensive and oral hypoglycemic agents were held. Blood glucose was controlled with insulin. The next morning, patient developed new onset ataxia and slurring of speech. Cerebellar signs were present. Monitoring was continued. The following day, the symptoms resolved and he became hemodynamically stable. Repeat ECG showed normal sinus rhythm with ventricular rate of 78 bpm (Fig. 3: ECG after 48 h of admission showed normal sinus rhythm with heart rate of 78 bpm, qrs = 100 ms, corrected QT (Rautaharju) = 423 ms). He was discharged home on third day of admission in good health.

## Discussion

The genera Rhododendron which belongs to Ericaceae family contains grayanotoxin which is also known as rhodotoxin or dromedotoxin or acetyandromedol. Grayanotoxin I is the major toxic isomer that is responsible for most of the symptoms [6]. The toxins are found in leaves, petals and nectar. There is an evidence of phenotypic correlation between toxin and herbivore defense [7]. The toxins have been studied in animal models as well. A study showed that the goats developed symptoms of poisoning when fed with rhododendron branches [8]. In an experiment, pigs also showed similar results [9]. 

Grayanotoxins bind to voltage gated sodium channels. This binding stabilizes the channel’s activated (open) state. This leads to increased sodium permeability and persistent depolarization in excitable tissues like brain, heart, gastrointestinal tract and muscles [10]. This action is similar to that of aconite poisoning. Due to their shared mechanism, both toxins produce comparable effects. Aconite is also found in higher altitude like Rhododendron [11]. However, appearance of plant and its flower can easily help to distinguish them. Poisoning presents with symptoms involving multiple organs; cardiac (bradycardia, hypotension), CNS (altered mental status, seizure), respiratory (shortness of breath, chest tightness), muscular (tremors, weakness) [5]. 

A case was reported in rural Nepal in a man presenting with dizziness and bradycardia after consuming dried white rhododendron flowers [6]. Similar case was also reported in Manang after consumption of wild honey consuming grayanotoxin [12]. Consumption of honey imported from Turkey developed similar symptoms in a patient in US [4]. Poon W et al. also reported a case of grayanotoxin poisoning in infants from Hongkong [13]. However, poisoning following ingestion of fresh flower has not been reported.

Mainstay of treatment for poisoning with rhododendron is supportive therapy. IV fluids for hypotension, atropine for bradycardia and occasionally vasopressors are required to maintain blood pressure. Prognosis is good and patients usually recover fully within 24 to 48 h [6, 11]. In this case, patient well responded to iv fluid bolus. Hence, Atropine and vasopressors were not considered.

Based on these findings, patient presenting with bradycardia, hypotension, and chest discomfort in the background of consumption of plants or plant products in high altitude, rhododendron intoxication should be suspected. However, not all species of rhododendron are toxic. Thus, laboratory evaluation is needed to confirm the presence of toxin in vivo and in vitro. In this case, laboratory confirmation was not possible due to unavailability of resources. The case was reported to Public Health Office, Dolakha.

WHO recommends established center to be provided expertise to manage the cases of poisoning. Prevention of poisoning is one of the important indicators of sustainable development goals [14]. It is vital to create public awareness regarding consumption of rhododendron products. This also calls for training needs for health care providers to manage locally relevant toxicity.

## Conclusion

Rhododendron intoxication, primarily caused by grayanotoxins, typically presents with bradycardia, hypotension, and neurological disturbances. Though rare, it calls for high clinical suspicion particularly in regions where rhododendron or its products are consumed. Early recognition and supportive treatment usually lead to complete recovery within 24 to 48 h. Considering the potential severity, raising public awareness and enhancing local diagnostic and management capabilities play cardinal role for effective prevention and control of such poisoning cases.

## Data Availability

No datasets were generated or analysed during the current study.

## Abbreviations

GCS: Glasgow coma scale
ECG: Electrocardiogram
AMS: Acute mountain sickness
DKA: Diabetic ketoacidosis
WBC: White blood counts
Na: Sodium
K: Potassium
AST: Aspartate transaminase
ALT: Alanine transaminase
gm/dl: grams per deciliter
mg/dl: milligrams per deciliter
mEq/L: milli equivalents per liter
IU/L: International units per liter
ml: milliliters
mmHg: millimeters of mercury
bpm: beats per minute
IV: intravenous
WHO: World Health Organization

## References

[1] Sharma A, Patel S, Maiti A, Mishra A. An epidemiological study of poisoning: a follow-up study from a tertiary care hospital. J Prim Care Spec. 2024;5(1):54.

[2] datadot [Internet]. [cited 2025 May 27]. 84FD3DE. [Cited 2025 May 24] Available from: https://data.who.int/indicators/i/55CF708/84FD3DE

[3] Database N, Nepal D. 2023 [cited 2025 May 27]. Rhododendron: National Flower of Nepal. [Cited 2025 May 25] Available from: https://www.nepaldatabase.com/rhododendron-national-flower-of-nepal

[4] DiSalvo P, Khorolsky C, Filigenzi M, Poppenga R, Hoffman RS. Confirmed grayanotoxin poisoning with bradycardia from a gift of imported honey. J Emerg Med. 2022;63(2):e45–8. 35871991 10.1016/j.jemermed.2022.05.009

[5] Gunduz A, Turedi S, Russell RM, Ayaz FA. Clinical review of grayanotoxin/mad honey poisoning past and present. Clin Toxicol. 2008;46(5):437–42. 10.1080/15563650701666306 18568799

[6] Baral S, Baral BK, Sharma P, Shrestha SL. Dried rhododendron flower ingestion presenting with bradycardia and hypotension: a case report. J Med Case Rep. 2022;16(1):189. 35551667 10.1186/s13256-022-03413-8 PMC9101929

[7] Fattorini R, Egan PA, Rosindell J, Farrell IW, Stevenson PC. Grayanotoxin I variation across tissues and species of rhododendron suggests pollinator-herbivore defence trade-offs. Phytochemistry. 2023;212:113707. 37149121 10.1016/j.phytochem.2023.113707

[8] Puschner B, Holstege DM, Lamberski N. Grayanotoxin poisoning in three goats. J Am Vet Med Assoc. 2001;218(4):573. 11229512 10.2460/javma.2001.218.573

[9] Pischon H, Petrick A, Müller M, Köster N, Pietsch J, Mundhenk L. Grayanotoxin I intoxication in pet pigs. Vet Pathol. 2018;55(6):896–9. 30071802 10.1177/0300985818789482

[10] Lee SW, Choi SH, Hong YS, Lim SI. Grayanotoxin poisoning from flower of *rhododendron mucronulatum* in humans. Bull Environ Contam Toxicol. 2007;78(2):132–3. 17401506 10.1007/s00128-007-9053-6

[11] Cardiovascular effects of poisoning following ingestion of. dried Rhododendron flower in a middle-aged woman: a case report | Bhutan Sorig Journal [Internet]. [cited 2025 May 26]. Available from: https://bsj.com.bt/index.php/bsj/article/view/108

[12] Adhikari A, Aryal R, Baral P, Acharya B, Neupane P, Paudel A, et al. Wild honey grayanotoxin intoxication in rural Himalayan region of nepal: a case report. Ann Med Surg 2012. 2025;87(3):1667–9. 10.1097/MS9.0000000000002877 PMC11981317 40213180

[13] Poon WT, Ho CH, Yip KL, Lai CK, Cheung KL, Sung RYT, et al. Grayanotoxin poisoning from rhododendron Simsii in an infant. Hong Kong Med J Xianggang Yi Xue Za Zhi. 2008;14(5):405–7. 18840915

[14] Prevention and management. of cases of poisoning [Internet]. [cited 2025 May 26]. Available from: https://www.who.int/teams/environment-climate-change-and-health/chemical-safety-and-health/incidents-poisonings/prevention-and-management-of-cases-of-poisoning

